# Easy, reproducible extraperitoneal pelvic access for robot - assisted radical prostatectomy

**DOI:** 10.1590/S1677-5538.IBJU.2018.0175

**Published:** 2019

**Authors:** Antonio Rebello Horta Gorgen, Christian P. Pavlovich

**Affiliations:** 1 Hospital de Clínicas de Porto Alegre, Porto Alegre, Brasil;; 2 Brady Urological Institute, Johns Hopkins University School of Medicine, Baltimore, Maryland, United States

## Abstract

Robot - assisted radical prostatectomy is commonly performed transperitoneally (tRARP), although the extraperitoneal (eRARP) approach is a safe and effective alternative that may be preferred in certain situations. We developed a novel method of direct access into the space of Retzius with a visual obturator port (Visiport™) for laparoscopic or robotic prostatectomy.

We present an instructional video of extraperitoneal pelvic access for eRARP with both internal and external camera views. The patient is first placed in lithotomy and 15° Trendelenburg position. The camera is inserted infraumbilically and angled caudally. The pre-peritoneal space is accessed through the anterior rectus fascia using a Visiport™ (Covidien, $ 60 www.esutures.com), and the working space is developed with a kidney - shaped balloon OMSPDBS2™ (Covidien, $ 49 www.esutures.com). After the space is insufflated, subsequent trocars are angled in extraperitoneally under direct vision. The average time from incision to final port placement after a learning curve of about 50 cases is 8 minutes (IQR 7-10).

We have performed over 1.000 cases using this technique and eRARP has become our procedure of choice. Our last 500 + cases were performed robotically. Approximately 10% of the time peritoneotomies were noted, but rarely did these require conversion to tRARP. There have been no bowel or other abdominal organ injuries, major vascular or other complications in any of these cases.

## ARTICLE INFO

**Available at: http://www.intbrazjurol.com.br/video-section/20180175_Gorgen_et_al**

**Int Braz J Urol. 2019; 45 (Video #1): 189-189**

